# Supplementary material for: Impacts of Sodium Arsenite on Wood Microbiota of Esca-Diseased Grapevines
Source: J Fungi (Basel). 2021 Jun 22;7(7):498. doi: 10.3390/jof7070498 (PMC8304700; doi:10.3390/jof7070498)
Supplement: Supplementary file 1 [file jof-07-00498-s001.zip › jof-1255605-supplementary.pdf]

Fig S1. Non-metric multidimensional scaling (NMDS) analysis of fungal communities based on the Bray-Curtis dissimilarity index. Data used for this analysis contain OTUs > 10 sequences. Each point represents data from a single sample. nMDS of fungal communities from treated and untreated vines (A) of all regions (B), in 2014 and 2015 (C) in different types of tissue (D).

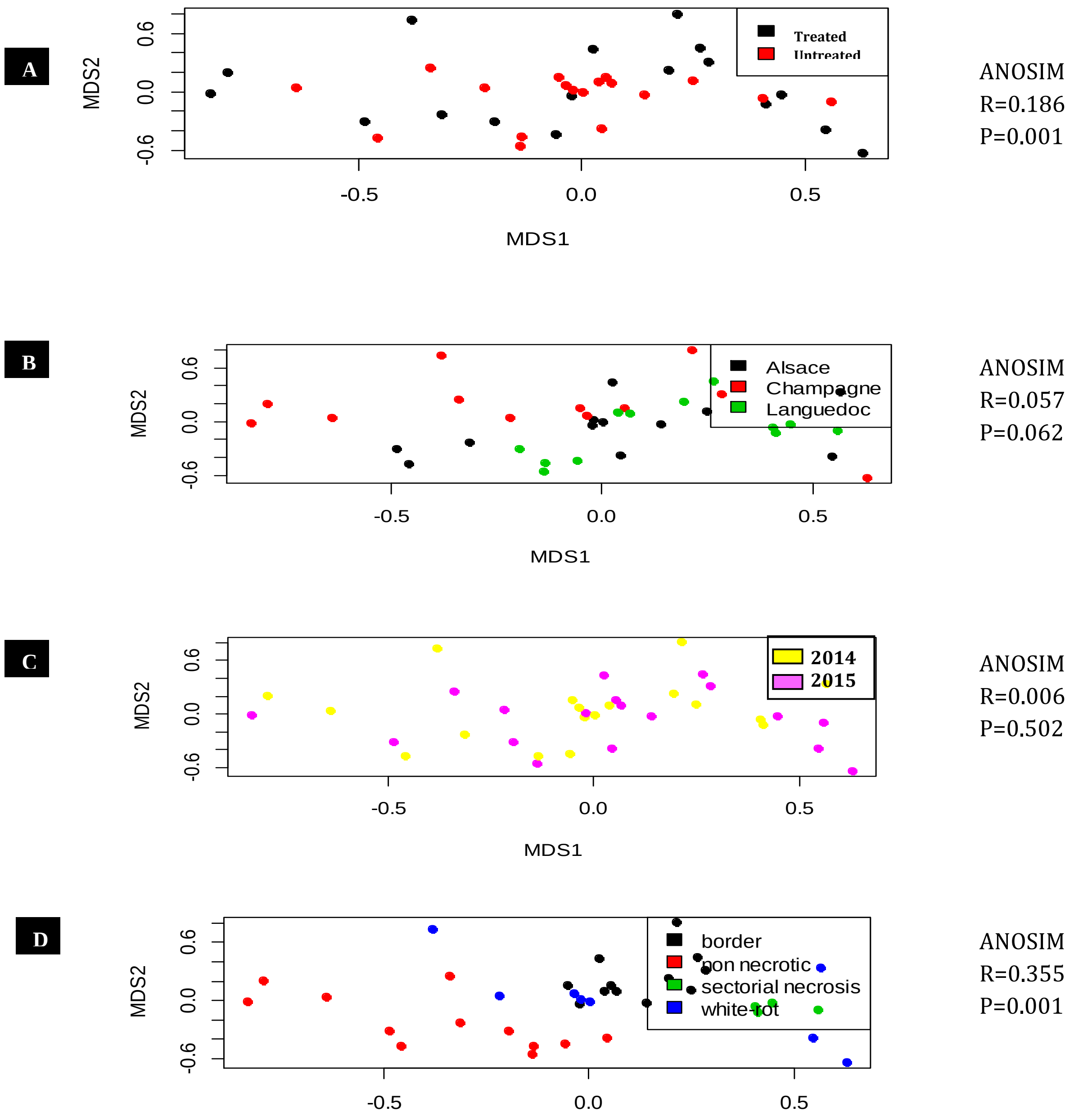

Figure S2. Non-metric multidimensional scaling (NMDS) analysis of fungal communities of untreated vines based on the Bray-Curtis dissimilarity index. Data used for this analysis contain all OTUs. Each point represents data from a single sample. Data used for this analysis contain all OTUs. nMDS of fungal communities from three regions (A) and from different types of tissues (B).

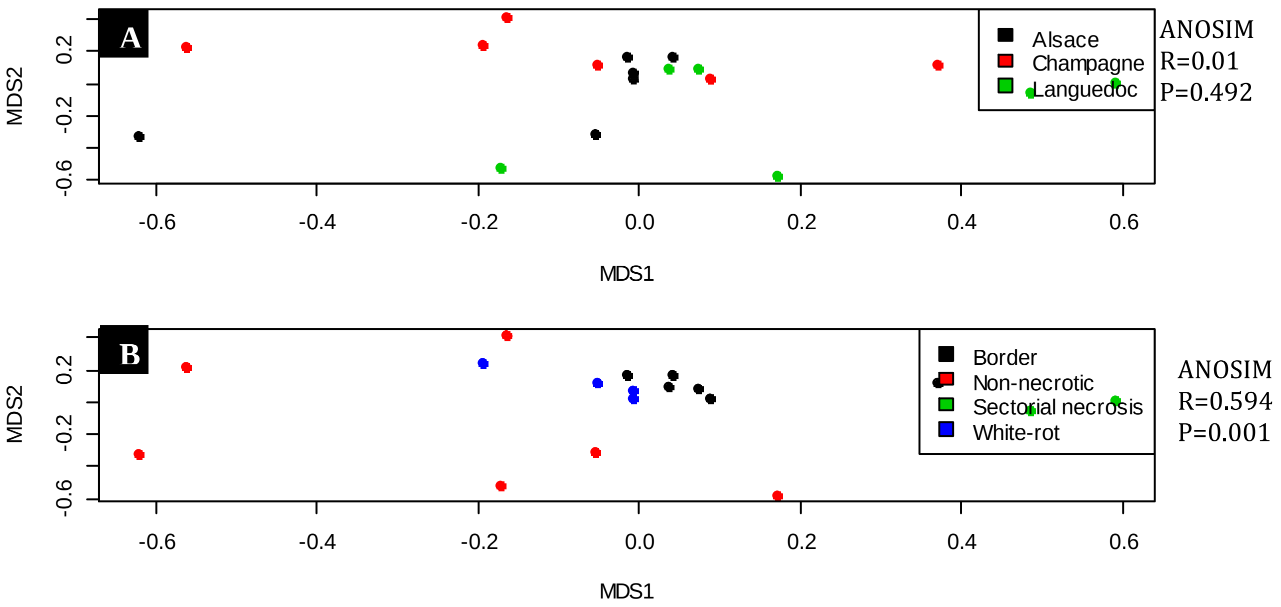

Figure S3. Non-metric multidimensional scaling (NMDS) analysis of bacterial communities of untreated vines based on the Bray-Curtis dissimilarity index. Data used for this analysis contain all OTUs. Each point represents data from a single sample. Data used for this analysis contain all OTUs. nMDS of fungal communities from three regions (A) and from different types of tissues (B).

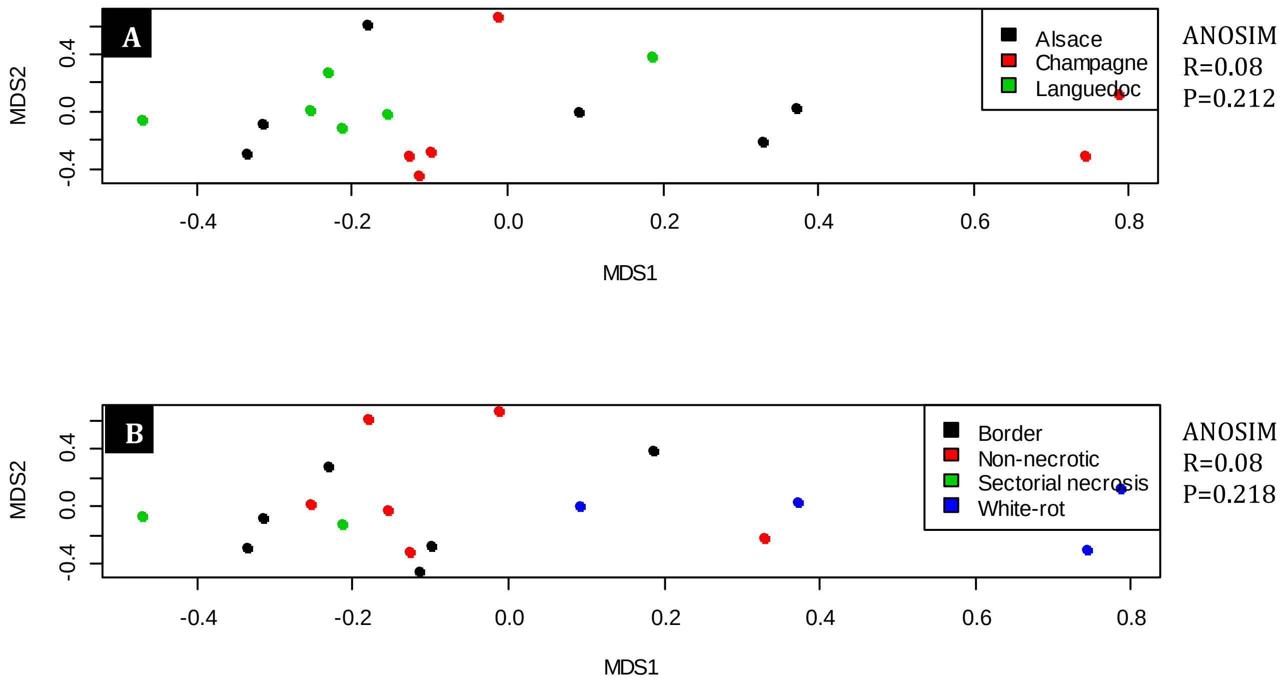

Table S1. Number of fungal and bacterial OTUs and reads per sample.

Region	Woody tissues	Year	OTUs number Bacteria	Number of reads
Alsace	non necrotic	2014	905	269102
Alsace	non necrotic	2015	1274	26607
Champagne	non necrotic	2014	1051	169588
Champagne	non necrotic	2015	1341	56452
Languedoc	non necrotic	2014	1531	67069
Languedoc	non necrotic	2015	1184	101447
Champagne	border	2014	787	66647
Champagne	border	2015	2090	119157
Alsace	border	2014	634	43405
Alsace	border	2015	1266	109393
Languedoc	border	2014	-	-
Languedoc	border	2015	1100	53123
Alsace	white-rot	2014	258	45720
Alsace	white-rot	2015	653	150876
Champagne	white-rot	2014	405	79056
Champagne	white-rot	2015	591	120819
Languedoc	sectorial necrosis	2014	1273	176102
Languedoc	sectorial necrosis	2015	1513	134858

“-“ : PCR did not work

Region	Woody tissues	Year	OTUs number Fungi	Number of reads
Alsace	non necrotic	2014	439	306871
Alsace	non necrotic	2015	389	77800
Champagne	non necrotic	2014	316	44178
Champagne	non necrotic	2015	370	88752
Languedoc	non necrotic	2014	387	53890
Languedoc	non necrotic	2015	348	53129
Champagne	border	2014	295	91815
Champagne	border	2015	249	53893
Alsace	border	2014	242	94211
Alsace	border	2015	319	89336
Languedoc	border	2014	300	96616
Languedoc	border	2015	335	103790
Alsace	white-rot	2014	230	35990
Alsace	white-rot	2015	256	72382
Champagne	white-rot	2014	178	16144
Champagne	white-rot	2015	239	57372
Languedoc	sectorial necrosis	2014	273	71638
Languedoc	sectorial necrosis	2015	263	48993

Table S2. Fungal strains isolated from grapevine woody tissues in 2015

	Region	Tissue	Arsenite
Phaeomoniella chlamydospora	Alsace	Black punctuation	treated
Fomitiporia mediterranea	Champagne	Border	untreated
Eutypa lata	Alsace	Sectorial necrosis	treated
Trichoderma sp.	Champagne	Black punctuation	treated
Penicillium sp. Strain 1	Champagne	Border	treated
Penicillium sp. Strain 2	Languedoc	Non-necrotic	treated

Table S3. Simpson and Shannon indexes of non-necrotic, border and white-rot tissues of Alsace, Champagne and Languedoc in 2014 and 2015.

Simpson (2014)	Alsace	Non-necrotic	Border	White rot
Treated	**Fungi**	0.42	0.11	0.62
	**Bacteria**	0.86	0.87	0.64
Untreated	**Fungi**	0.89	0.55	0.19
	**Bacteria**	0.70	0.98	0.95

Simpson (2014)	Champagne	Non-necrotic	Border	White rot
Treated	**Fungi**	0.79	0.68	0.43
	**Bacteria**	0.45	0.99	0.62
Untreated	**Fungi**	0.53	0.37	0.32
	**Bacteria**	0.09	0.98	0.84

Simpson (2014)	Languedoc	Non-necrotic	Border	Sectorial necrosis
Treated	**Fungi**	0.91	0.76	0.76
	**Bacteria**	0.98	-	0.87
Untreated	**Fungi**	0.91	0.45	0.67
	**Bacteria**	0.92	0.80	0.97

Simpson (2015)	Alsace	Non-necrotic	Border	White rot
Treated	**Fungi**	0.85	0.65	0.78
	**Bacteria**	0.99	0.84	0.59
Untreated	**Fungi**	0.69	0.60	0.24
	**Bacteria**	0.79	0.96	0.79

Simpson (2015)	Champagne	Non-necrotic	Border	White rot
Treated	**Fungi**	0.81	0.77	0.56
	**Bacteria**	0.98	0.98	0.76
Untreated	**Fungi**	0.74	0.36	0.13
	**Bacteria**	0.99	0.99	0.77

Simpson (2015)	Languedoc	Non-necrotic	Border	Sectorial necrosis
Treated	**Fungi**	0.84	0.64	0.79
	**Bacteria**	0.35	0.94	0.96
Untreated	**Fungi**	0.89	0.41	0.85
	**Bacteria**	0.98	0.69	0,99

Shannon (2014)	Alsace	Non-necrotic	Border	White rot
Treated	**Fungi**	1.04	0.43	1.53
	**Bacteria**	2.51	3.4	1.39
Untreated	**Fungi**	2.89	1.24	0.57
	**Bacteria**	1.73	4.98	4.40

Shannon (2014)	Champagne	Non-necrotic	Border	White rot
Treated	**Fungi**	2.64	1.43	1.17
	**Bacteria**	1.59	4.87	1.34
Untreated	**Fungi**	1.26	0.90	0.99
	**Bacteria**	0.37	5.24	2.37

Shannon (2014)	Languedoc	Non-necrotic	Border	Sectorial necrosis
Treated	**Fungi**	3.06	1.70	1.80
	**Bacteria**	5.40	-	3.27
Untreated	**Fungi**	3.19	1.01	1.60
	**Bacteria**	4.28	2.63	4.77

Shannon (2015)	Alsace	Non-necrotic	Border	White rot
Treated	**Fungi**	2.60	1.52	1.79
	**Bacteria**	5.94	3.39	1.59
Untreated	**Fungi**	2.14	1.57	0.66
	**Bacteria**	2.13	4.45	2.33

Shannon (2015)	Champagne	Non-necrotic	Border	White rot
Treated	**Fungi**	2.16	1.83	1.51
	**Bacteria**	5.50	5.51	1.97
Untreated	**Fungi**	2.38	0.92	0.44
	**Bacteria**	6.25	5.86	2.15

Shannon (2015)	Languedoc	Non-necrotic	Border	Sectorial necrosis
Treated	**Fungi**	2.73	1.36	2.03
	**Bacteria**	1.60	4.52	4.48
Untreated	**Fungi**	3.17	0.94	2.21
	**Bacteria**	5.36	1.93	5.51

Table S4. Statistical results of the Kruskal-Wallis tests for the 20 most abundant fungal species. In yellow, the statistics showed significant differences.

Kruskal_Wallis test (non parametrique)	*Alternaria spp.*	*Aspergillus spp.*	*Aureobasidium pullulans*	*Unknown Agaricales genus*	*Cladosporium spp.*	*Diplodia seriata*	*Eutypa lata*	*Fomitiporia mediterranea*	*Hypocrea lixii*	*Ionotus hispidus*	*Lepiota brunneoincarnata*	*Mucor circinelloides.*	*Mycena maurella*	*Penicillium spp.*	*Phaeoacremonium viticola*	*Phaeoacremonium chlamydosporum*	*Phaeoacremonium fraxinopennsylvanicum*	*Sebacina vermifera*	*Seimatosporium vitis*	*Trichoderma atroviride*
Tissues	0.03 (Non-necrotic tissue difer from the other)	0,472	0.05 (Non-necrotic tissue difer from the other)	0,3491	0,043	0.018 (sectorial necrosis differ from other)	0,594	0,9182	0,238	0,453	0,453	0,75	0,88	0,632	0,147	0.006 (border from the other tissue)	0,499	0,616	0,1122	0,702
Region	0,205	0,125	0,737	0,07	0,519	0,09	0,082	0,894	0,28	0,441	0,86	0,663	0,894	0,352	0.004 (Languedoc from the orther region)	0,325	0,472	0,6	0,035	0,47
Treated/Untreated	0,75	0,07	0,964	0,757	0,825	0,269	0,08	<0.001	0,401	0,026	0,03	0,543	0,02	0,475	0,691	0,894	0,724	0,5658	0,02	0,233

Table S5. Statistical results of the Kruskal-Wallis tests for the 20 most abundant bacterial genera. In yellow, the statistics showed significant differences.

Kruskal-Wallis test	*Amnibac-terium sp.*	*Badyrhi-zobium sp.*	*Brevun-dimonas sp.*	*Buttiau-xella sp.*	*Cellulo-monas sp.*	*Chryseoba cterium sp.*	*Cloac-bacterium* sp.	*Curto-bacterium* sp.	*Entero-bacter sp.*	*Erwinia* sp.	*Methylo-bacterium* sp.	*Micro-bacterium* sp.	*Noc-ardia sp.*	*Pantoea* sp.	*Pseudo-monas* sp.	*Raoultella* sp.	*Rhizobium* sp.	*Sodalis* sp.	*Sphingo-monas* sp.	*Yersinia* sp.
Tissues	0.14	0.045	0.057	0.57	0.075	0.93	0.811	0.035	0.216	0.01	0.75	0.452	0.53	0.018	0.021	0.119	0.169	0.093	0.042	0.038
Region	0.125	0.1022	0.2351	0.486	0.573	0.9623	0.7545	0.064	0.732	0.3976	0.562	0.3874	0.149	0.103	0.703	0.593	0.83	0.5521	0.302	0.24
Treated/ Untreated	0.163	0.501	0.63	0.634	0.148	0.809	0.923	0.336	0.531	0.847	0.652	0.847	0.441	0.847	0.923	0.289	0.93	0.136	0.847	0.386

Table S6. ITS1 sequences of the 20 most abundant fungal OTUs identified in grapevine trunk.
In blue 18S, in purple 5.8S

#OTU-1 *Alternaria spp*. from to the *A. alternata* clade
AAGTCGTAACAAGGTCTCCGTAGGTGAACCTGCGGAGGGATCATTACACAAATATGAAGGCGGGCTGGAACCTCTCGGGGTTACAGCCTTGC
TGAATTATTCACCCTTGTCTTTTGCGTACTTCTTGTTTCCTTGGTGGGTTCGCCCACCACTAGGACAAACATAAACCTTTTGTAATTGCAATCAG
CGTCAGTAACAAATTAATAATTACAACTTTCAACAACGGATCTCTTGGTTCTGGCATCGATGAAGAACGCAGC

#OTU-2 *Aspergillus spp.* from to the *A. penicillioides* clade
AAGTCGTAACAAGGTTTCCGTAGGTGAACCTGCGGAAGGATCATTACCGAGTGTTTGGGCGCCCCTTCTCGGGGTGTCCGTCCTCCCATCCGT
GTCTCTTTGTACCCTGTTGCTTCGGCGGGCCCGCCCTCCGTGGCCGCCGGGGGGCCTCCCTGGCCcccGGGCCCGTGCCCGCCGGAGACCTCAA
CCATGAACACTGTCTGAAGGTTGCAGTCTGAGTACCGATAGTAAAAATCGTTAAAACTTTCAACAACGGATCTCTTGGTTCCGGCATCGATGA
AGAACGCAGC

#OTU-3 *Aurebasidium pullulans*
AAGTCGTAACAAGGTTTCCGTAGGTGAACCTGCGGAAGGATCATTAAAGAGTAAGGGTGCTCAGCGCCCGACCTCCAACCCTTTGTTGTTAAA
ACTACCTTGTTGCTTTGGCGGGACCGCTCGGTCTCGAGCCGCTGGGGATTCGTCCCAGGCGAGCGCCCGCCAGAGTTAAACCAAACTCTTGTT
ATTTAACCGGTCGTCTGAGTTAAAATTTTGAATAAATCAAAACTTTCAACAACGGATCTCTTGGTTCTCGCATCGATGAAGAACGCAGC

#OTU-4 Unknown *Agaricales* genus
AAGTCGTAACAAGGTTTCCGTAGGTGAACCTGCGGAAGGATCATTATTGAATGTATTTGCAAGTTGGTTGTAGCTGGCTTCTCGGAGTATTGT
GCTCGCCACTTGTCTCTACTACTTTTCCACCTGTGAACCTTTGTAGATCACGAATGAACTCTCGCCTGAAAATGGCGGTTTGAGGGACTTGTCC
CTTGATAGTTCGTGGTCTATGTTTATATCTACCCTATGTTTGTCTTTGAATGTCTTTTAAATGGTCTTTGACCTTTAAATACTATACAACTTTTAG
CAACGGATCTCTTGGCTCTCGCATC

#OTU-5 *Cladosporium spp.*
AAGTCGTAACAAGGTCTCCGTAGGTGAACCTGCGGAGGGATCATTACAAGTGACCCCGGTCTAACCACCGGGATGTTCATAACCCTTTGTTGT
CCGACTCTGTTGCCTCCGGGGCGACCCTGCCTTCGGGCGGGGGCTCCGGGTGGACACTTCAAACTCTTGCGTAACTTTGCAGTCTGAGTAAAC
TTAATTAATAAATTAAAACTTTTAACAACGGATCTCTTGGTTCTGGCATCGATGAAGAACGCAGC

#OTU-6 *Diplodia seriata*

AAGTCGTAACAAGGTTTCCGTAGGTGAACCTGCGGAAGGATCATTACCGAGTTCTCGGGCTTCGGCTCGAATCTCCCACCCTTTGTGAACATACCTCTGTTGCTTTGGCGGCTCTTTGCCGCGAGGAGGCCCTCGCGGGCCCCCCGCGCGCTTTCTGCCAGAGGACCTTCAAACTCCAGTCAGTAAACGTCGACGTCTGATAAACAAGTTAATAAACTAAAACTTTCAACAACGGATCTCTTGGTTCTGGCATCGATGAAGAACGCAGC

#OTU-7 *Eutypa lata*

AAGTCGTAACAAGGTCTCCGTTGGTGAACCAGCGGAGGGATCATTACAGAGTTACCTAACTCCAAACCCATGTGAACTTACCTATGTTGCCTCGGCGGGGAAGCCTACCCGGTACCTACCCTGTAGCTACCCGGGAGCGAGCTACCCTGTAGCCCGCTGCAGGCCTACCCGCCGGTGGACACTTAAACTCTTGTTTTTTTAGTGATTATCTGAGTGTTTATACTTAATAAGTTAAAACTTTCAACAACGGATCTCTTGGTTCTGGCATCGATGAAGAACGCAGC

#OTU-8 *Fomitiporia mediterranea*

AAGTCGTAACAAGGTTTCCGTAGGTGAACCTGCGGAAGGATCATTAACGAGTTGGAACGTGGAGGTTGATGCTGGTGCATatataGTGTACATGTGCTCGCCTTCACACTCTTCATCCACTCAACCCCTGTGCACTTTATCAGAGTTAGTAATAGTATTGTGGTGGCAGCCGTTTGTTATTCATTGTTAGAAGCGGGGTAACTCTTTCTAGCAGTAGTAATAATAACAATCTTGGTTCTACTACTATTACTGTGAACACTTTGACTTTTACTTATACAAACACTTTGCTTGTTCTTGTGAATGTGTAATGCTCCTTGTGAGCGAAATACAAATATACAACTTTCAACAACGGATCTCTTGGCTCTCGCATCGATGAAGAACGCAGC

#OTU-9 *Hypocrea lixii (syn Trichoderma lixii)*

AAGTCGTAACAAGGTCTCCGTTGGTGAACCAGCGGAGGGATCATTACCGAGTTTACAACTCCCAAACCCAATGTGAACGTTACCAAACTGTTGCCTCGGCGGGATCTCTGCCCCGGGTGCGTCGCAGCCCCGGACCAAGGCGCCCGCCGGAGGACCAACCTAAAACTCTTATTGTATACCcccTCGCGGGTTTTTTTATAATCTGAGCCTTCTCGGCGCCTCTCGTAGGCGTTTCGAAAATGAATCAAAACTTTCAACAACGGATCTCTTGGTTCTGGCATCGATGAAGAACGCAGC

#OTU-10 *Ionotus hispidus*

AAGTCGTAACAAGGTTTCCGTAGGTGAACCTGCGGAAGGATCATTATCGAGTTTTGAAGCCGGAGGCCTGTGCTGGTGCGGAAACGCACATGTGCACGGCTTTCGTGCTCAAATCCATTTCAAACCCCTGTGCACTTTTGAACCGGTTGAAGTTAGTAGTGAGTAACACTTTCGACTTGCGACCTAGTACTGCGAGTAATACTAGTAGGAGGGTCTTTTGAGGCCTTTCGAACGTTTGAAAGCGGGAAAACGTTAAGAGAAGAGAGGGTGAAGGCGAGCGCTTTGACTAGTTTGTATTACAAACCCTTTTATTGTTATGTGAATGTAATGCTCCTTGTGGGCGATAATTTGTACAACTTTCAACAACGGATCTCTAGGCTCTCGCATCGATGAAGAACGCAGC

#OTU-11 *Lepiota brunneoincarnata*
AAGTCGTAACAAGGTTTCCGTAGGTGAACCTGCGGAAGGATCATTATTGAATAAACTTGGTGGGTTGTTGCTGGCTTCTTGGAGCATGTGCAC
GCTCATCGACTTTATCCATCCACCTGTGCACCTTCTGTAGTCTTTGAAATGAAAGCGGCTGAGCCTCGATGGGCATTTTGCCCTATCGGATGTG
AGGAATGCTTTTGTGAAGGCATGGCTCTCCTCAAAGGCCTGTGATCGTTTCTTGGACTATGTTtttCCATATACCACATAGCATGTTGTAGAATGT
ATCGGTGGGCCTCTGTGCCTATAGAACTCAATACAACTTTCAGCAACGGATCTCTTGGCTCTCGCATCGATGAAGAACGCAGC

#OTU-12 *Mucor circinelloides*
AAGTCGTAACAAGGTTTCCGTAGGTGAACCTGCGGAAGGATCATTAAATAATCAATAATTTTGGCTTGTCCATTATTATCTATTTACTGTGAAC
TGTATTATTACTTGACGCTTGAGGGATGCTCCACTGCTATAAGGATAGGCGGTGGGGATGTTAACCGAGTCATAGTCAAGCTTAGGCTTGGTA
TCCTATTATTATTTACCAAAAGAATTCAGAATTAATATTGTAACATAGACCTAAAAAATCTATAAAACAACTTTTAACAACGGATCTCTTGGTT
CTCGCATCGATGAAGAACGCAGC

#OTU-13 *Mycena maurella*
AAGTCGTAACAAGGTTTCCGTAGGTGAACCTGCGGAAGGATCATTACTGAATAACTGGAGTGTTGTAGCTGGCTTTTCGGAGCATGTGCACGC
ATTTCAAATTCATCTTTACCACCTGTGCACTTTTGTAGACTGTGATAACTCTCAATGGTTTTTCCGTTGGATTGAAGGACTTGCGTCTAGCTGTC
TTTCGATTTCACAGTCTATGTCTCATCTACTATAAAAAGTCTAGAATGTAACTTGTGGGTCTTTTGACCTATAAAACTTATACAACTTTCAACA
ACGGATCTCTTGGCTCTCGCATCGATGAAGAACGCAGC

#OTU-14 *Penicillium spp.* from to the *P. glabrum* clade
AAGTCGTAACAAGGTTTCCGTAGGTGAACCTGCGGAAGGATCATTACTGAGTGAGGGCCCTCTGGGTCCAACCTCCCACCCGTGTTTATTGTA
CCTTGTTGCTTCGGTGCGCCCGCCTCACGGCCGCCGGGGGGCTTCTGCCCCCGGGTCCGCGCGCACCGGAGACACTATTGAACTCTGTCTGAA
GATTGCAGTCTGAGCATAAACTAAATAAGTTAAAACTTTCAACAACGGATCTCTTGGTTCCGGCATCGATGAAGAACGCAGC

#OTU-15 *Phaeocremonium viticola*
AAGTCGTAACAAGGTCTCCGTTGGTGAACCAGCGGAGGGATCATTACAGAGTTTCATACTCCAAACCCTTTGTGAACATACCTGTTTCCGTTG
CTTCGGCAGGTCGGGGGTCACCCCCCGCCCGCCGCCGGGCCCCCCTCGCGGGGTCGCCCGGCGGGCCTGCCGGAGGGCACAAGACTCTGTAT
TTCAAAACTACCTCTCTGAGTTATCTTTACAAATAAGTAAAAACTTTCAACAACGGATCTCTTGGTTCTGGCATCGATGAAGAACGCAGC

#OTU-16 *Phaeomoniella chlamydospora*

AGTCGTAACAAGGTTTCCGTAGGTGAACCTGCGGAAGGATCATTATCGAGTCAGGGTCCTCTGGGCCCGATCTCCAACCCTTTGTTTATCATA
CCTTTGTTGCTTTGGCAGACCCGTCCTTCGGACCGTCGGGGGCGTTCAGTCGCCTCTGGCCAGCGTCTGCCAGTAGCCCAACCAAAATTCTTT
GTTACATGTGACGTCTGAACGGTTCCATCAAAATCAAACCAAAACTTTCAACAACGGATCTCTTGGTTCTGGCATCGATGAAGAACGCAGC

#OTU-17 *Phaeoacremonium fraxinopennsylvanicum*
AAGTCGTAACAAGGTCTCCGTTGGTGAACCAGCGGAGGGATCATTACCGAGTCTCGTACTCCAAACCCTTTGTGAACATACCTGTTTCGTTGC
TTCGGCAGGCCCGGGGGTCACTCCCCGGCCCGCCGCCGGCACCCCCTCGCCGGGGCGCATGGCGGGCCTGCCGGAGGGCACAGACTCTGTAT
TCAAAAACGTACCTCTCTGAGTTATCTTTACAAACAAGTAAAAACTTTCAACAACGGATCTCTTGGTTCTGGCATCGATGAAGAACGCAGC

#OTU-18 *Sebacina* spp.
AAGTCGTAACAAGGTTTCCGTAGGTGAACCTGCGGAAGGATCATTAATGAACTTCAAGTCGGTCGTCTGTGCTGGTGGCAACACATGTGCACG
TCGGCTGCAAATCCACACACCTGTGCACCTTTGACTTTGGGATCCGCTGGTTGGCTTGTCCGACTTGCAGCTGGTTGACTTGTGTGTCGACCTG
GGTCTCAGGGTATTTTTACACATACTGAATGTAACGGAATGTCACTGTGCATAATGCGCATAATAACAACTTTCAACAACGGATCTCTTGGCTC
TCGCAT

#OTU-19 *Seimatosporium vitis*
AAGTCGTAACAAGGTCTCCGTTGGTGAACCAGCGGAGGGATCATTACAGAGTTATCTAACTCCCAAACCCATGTGAACTTACCATTGTTGCCT
CGGCAGAACCTACCCGGTACCTACCCTGTAACGACCTACCCTGTAGCGAGTTACCCGGGAACGGCCTACCCTGTAGCGCGCTGCCGGTGGACT
TCTAAACTCTTGTTATTTATTGTAATCTGAGCGTCTTATTTTAATAAGTCAAAACTTTCAACAACGGATCTCTTGGTTCTGGCATCGATGAAGA
ACGCAGC

#OTU-20 *Trichoderma atroviride*
AAGTCGTAACAAGGTCTCCGTTGGTGAACCAGCGGAGGGATCATTACCGAGTTTACAACTCCCAAACCCAATGTGAACCATACCAAACTGTTG
CCTCGGCGGGGTCACGCCCCGGGTGCGTCGCAGCCCCGGAACCAGGCGCCCGCCGGAGGGACCAACCAAACTCTTTTCTGTAGTCCCCTCGCG
GACGTTATTTCTTACAGCTCTGAGCAAAAATTCAAAATGAATCAAAACTTTCAACAACGGATCTCTTGGTTCTGGCATCGATGAAGAACGCAG
C

Table S7. NCBI best BLASTN hits of the ITS1 sequences from the 20 most abundant fungal OTUs identified in grapevine trunk.

OTU	Best Hit NCBI	ID NCBI	Query coverage	E value	Identity %
1	*Alternaria spp.* from to the *A. alternata* clade	MT556702.1	100	1,00E-131	100
2	*Aspergillus spp.* from to the *A. penicillioides* clade	KF800460.1	100	3,00E-148	100
3	*Aurebasidium pullulans*	MT573468.1	100	7,00E-140	100
4	Unknown *Agaricales genus*	LC205406.1	100	6,00E-131	99
5	*Cladosporium spp.*	MT852065.1	100	1,00E-126	100
6	*Diplodia seriata*	MT587389.1	100	4,00E-137	100
7	*Eutypa lata*	MN433694.1	100	7,00E-145	100
8	*Fomitiporia mediterranea*	AY780427.1	100	0	100
9	*Hypocrea lixii*	KY554996.1	100	4,00E-152	100
10	*Ionotus hispidus*	FR686562.1	96	0,00E+00	99
11	*Lepiota brunneoincarnata*	KT002153.1	100	0	99
12	*Mucor circinelloides*	MG554336.1	100	3,00E-154	99
13	*Mycena maurella*	JF908413.1	100	8,00E-160	99
14	*Penicillium spp.* from to the *P. glabrum* clade	MT441616.1	100	5,00E-136	100
15	*Phaeocremonium viticola*	MN209190.1	98	4,00E-137	100
16	*Phaeomoniella chlamydospora*	KP887017.1	100	2,00E-141	100
17	*Phaeoacremonium fraxinopennsylvanicum*	MH862855.1	99	2,00E-139	100
18	*Sebacina spp.*	EU910901.1	95	1,00E-127	96
19	*Seimatosporium vitis*	NR_156595.1	98	2,00E-144	100
20	*Trichoderma atroviride*	AF456917.1	100	4,00E-144	100
